# Characterization and Potential Applications of a Selenium Nanoparticle Producing and Nitrate Reducing Bacterium *Bacillus oryziterrae* sp. nov.

**DOI:** 10.1038/srep34054

**Published:** 2016-09-28

**Authors:** Peng Bao, Ke-Qing Xiao, Hui-Jiao Wang, Hao Xu, Peng-Peng Xu, Yan Jia, Max M. Häggblom, Yong-Guan Zhu

**Affiliations:** 1 Key Lab of Urban Environment and Health, Institute of Urban Environment, Chinese Academy of Sciences, Xiamen, 361021, P. R. China; 2 Ningbo Urban Environment Observation and Research Station, Chinese Academy of Sciences, Ningbo 315800, China; 3 State Key Lab of Urban and Regional Ecology, Research Center for Eco-Environmental Sciences, Chinese Academy of Sciences, Beijing 100085, P. R. China; 4 University of Science and Technology, Beijing 100083, P. R. China; 5 Beijing Fixgene Techology Co., Ltd, P. R. China; 6 National Engineering Laboratory for Hydrometallurgical Cleaner Production Technology, Institute of Process Engineering, Chinese Academy of Sciences, P. R. China; 7 Rutgers University, Department of Biochemistry and Microbiology, School of Environmental and Biological Sciences, 76 Lipman Drive, New Brunswick, NJ 08901, USA

## Abstract

A novel nitrate- and selenite reducing bacterium strain ZYK^T^ was isolated from a rice paddy soil in Dehong, Yunnan, China. Strain ZYK^T^ is a facultative anaerobe and grows in up to 150, 000 ppm O_2_. The comparative genomics analysis of strain ZYK^T^ implies that it shares more orthologues with *B*. *subtilis subsp*. *subtilis* NCIB 3610^T^ (ANIm values, 85.4–86.7%) than with *B*. *azotoformans* NBRC 15712^T^ (ANIm values, 84.4–84.7%), although *B*. *azotoformans* NBRC 15712^T^ (96.3% 16S rRNA gene sequence similarity) is the closest *Bacillus* species according to 16S rRNA gene comparison. The major cellular fatty acids of strain ZYK^T^ were iso-C_14:0_ (17.8%), iso-C_15:0_ (17.8%), and C_16:0_ (32.0%). The polar lipid profile consisted of phosphatidylethanolamine, diphosphatidylglycerol, phosphatidylglycerol and an unidentified aminophospholipid. Based on physiological, biochemical and genotypic properties, the strain was considered to represent a novel species of the genus *Bacillus*, for which the name *Bacillus oryziterrae* sp. nov. is proposed. The type strain is ZYK^T^ (=DSM 26460^T^ =CGMCC 1.5179^T^). Strain ZYK^T^ can reduce nitrate to nitrite and ammonium and possesses metabolic genes for nitrate reduction including *nar*, *nap* and *nrf*. Biogenic selenium nanoparticles of strain ZYK^T^ show a narrow size distribution and agree with the gaussian distribution. These selenium nanoparticles show significant dose-dependent inhibition of the lung cancer cell line H157, which suggests potential for application in cancer therapy.

Rice is widely cultivated and feeds more than half of the world’s population^1^. Paddy soils typically undergo wet and dry cycles during rice cultivation^2^ and diverse bacterial groups are active and play key roles in biogeochemical cycling, thus influencing the levels of trace elements, such as Se, in rice^3^,^4^. Members of the genus *Bacillus* are numerically dominant proteolytic bacteria in organic-matter amended paddy soils at different stages of rice development (e.g., vegetative growth, maximal tilling, and harvest stage)^5^. *Bacillus* spp. are crucial in both organic-nitrogen and inorganic-nitrogen transformation because of their high extracellular protease and nitrate reduction ability^6^. In paddy soils, *Bacillus* spp. may play an important role in the biological cycling of C and N, also because of their high resistance to a wet-dry rotation environment through endospore formation. Although dissimilatory nitrate reduction to ammonium (DNRA) in paddy soils has been well studied, *Bacillus* spp. responsible for this process were not considered until recently^7^.

Selenium is a key constituent of selenoproteins and is often lacking in rice-based diets where soils are Se-deficient and traditional rice diets do not provide Se levels adequate to maintain health^1^,^8^. Se availability in soils is of great importance in how this influences Se content in crops and consequently human Se status. Se concentrations in rice are strongly controlled by bioavailable Se in paddy soils, and water soluble and exchangeable Se is generally considered to be directly taken up by rice^9^,^10^. Hence, the cycling of Se in paddy soil is of considerable interest since the microbially mediated oxidation-reduction reactions of Se affects its bioavailability, mobility and solubility^11^,^12^,^13^. However little is known about the bacterial species involved and how they influence Se transformations. Bacterial reduction of selenite to elemental Se can result in selenium nanoparticles (SeNPs) precipitation and accumulation by binding to high-affinity proteins, including alkyl hydroperoxide reductase, peroxiredoxin, NADH peroxidase, and ribosomal protein^14^,^15^,^16^,^17^. Most SeNPs-bound proteins in bacteria are non-inducible selenite reduction-related oxidoreductases^17^. Artificial SeNPs have attracted increasing attention in the past decade because of their anticancer activities and low toxicity^18^,^19^. SeNPs were found to suppress the growth of prostate LNCaP cancer cells through Akt/Mdm2/AR controlled apoptosis^20^. Polyethylene glycol (PEG) is the most popular polymer for protein conjugation, and PEGylation has become a leading approach for overcoming most of the limits of the therapeutic application. PEG nanosized ultrasmall SeNPs overcome drug resistance in hepatocellular carcinoma HepG2 cells through induction of mitochondrial dysfunction^21^. Several synthetic techniques exist to produce SeNPs, however a major limitation is that they do not yield the narrow size distribution important for industrial applications. Biogenic SeNPs are more uniform in size than those from chemical processing and thus have better application potential.

We previously reported on the genome sequence of *Bacillus* sp. strain ZYK^T^^22^. Here, we provide additional details on its characteristics and phylogenetic position, indicating that the strain represents a new species of the genus *Bacillus*. Strain ZYK^T^ is a facultative anaerobic bacterium with the potential for dissimilatory nitrate reduction to ammonium, selenite reduction and consequent SeNPs production. Our analysis identifies the encoding genes that are responsible for DNRA in strain ZYK^T^. A proposed model is then established for the mechanisms of DNRA. Selenium transformation occurs through assembly of elemental selenium with intracellular high-affinity proteins. These biogenic SeNPs can significantly inhibit the non-small lung cancer cell line H157.

## Results

### Characterization of strain ZYK^T^

Here we provide a detailed taxonomic characterization indicating that stain ZYK^T^ represents a new species in the genus *Bacillus*. Phylogenetic relationships were inferred using the maximum-likelihood (ML) method (Supplementary Fig. S1), reconstructed using a selection of well-known *Bacillus* species, including *Bacillus azotoformans* NBRC 15712^T^ with 96.3% 16S rRNA gene sequence similarity, *Bacillus cohnii* DSM 6307^T^ with 95.2% 16S rRNA gene sequence similarity and *Bacillus subtilis* subsp. *subtilis* NCIB 3610^T^ with 93.0% gene sequence similarity, and additional species that are known for their ability to transform selenium^22^,^23^. Of these, *Bacillus selenitireducens* ATCC 700615^T^^24^ was the closest known selenite reducer and shared 90.0% 16S rRNA gene sequence similarity with strain ZYK^T^^22^.

Strain ZYK^T^ is a Gram-positive, spore-forming, motile, flagellated, bacilliform (0.2–0.3 μm wide and 1.5–2.0 μm long) rod (Supplementary Fig. S2)^22^. Strain ZYK^T^ is a facultative anaerobic bacterium and can grow in the presence of up to 15% O_2_ on agar plates. Strain ZYK^T^ grew between 21 and 45 °C, with an optimum temperature for growth at 30–40 °C. Growth at pH 6.0–7.6 was observed, with an optimum pH value of at about 7.0. Strain ZYK^T^ grew in the presence of NaCl concentrations ranging from 0% to 1.1% with an optimum near to 0% NaCl (Table 1)^22^. Strain ZYK^T^ was positive for nitrate and selenite reduction, and acid production from D-glucose and maltose. Strain ZYK^T^ was negative for acid production from D-mannose, D-mannitol and D-xylose, and hydrolysis of casein, starch, gelatin and aesculin, and can use L-histidine and tween-20 as carbon source. The DNA base composition was 36.1 mol% G+C, which is lower than compared to the phylogenetically most closely related *Bacillus* species (Table 1). This mol% G+C determined by the Tm technique matched that from genome analysis^22^. A detailed comparison of the characteristics of strain ZYK^T^ with *B*. *azotoformans*, *B*. *cohnii* and *B*. *subtilis* is presented in Table 1.

The fatty acid composition of strain ZYK^T^, *B*. *azotoformans* and *B*. *cohnii* are shown in Table 2. The major cellular fatty acids of strain ZYK^T^ were iso-C_14:0_ (17.8%), iso-C_15:0_ (17.8%), anteiso-C_15:0_ (10.4%), iso-C_16:0_ (10.5%) and C_16:0_ (32.0%), clearly separating it form *B*. *azotoformans* and *B*. *cohnii*. The polar lipid profile of strain ZYK^T^ contains PE, phosphatidylethanolamine; DPG, diphosphatidylglycerol; PG, phosphatidylglycerol; UPAL, and an unidentified aminophospholipid (Fig. S3a). The pattern of thin layer chromatogram of strain ZYK^T^ and *Bacillus azotoformans* were distinctly different in terms of UPL, unknown phospholipid and UL, unknown lipid (Fig. S3b).

### General genomic features and examples of gene family expansions

The strain ZYK^T^ genome shows large differences from the previously published genomes of *B*. *azotoformans* and *B*. *subtilis subsp*. *subtilis* NCIB 3610^T^ (Fig. S4a–c)^22^,^27^,^28^. Interestingly however, strain ZYK^T^ shared more orthologues with *B*. *subtilis subsp*. *subtilis* NCIB 3610^T^ than with *B*. *azotoformans*, although *B*. *azotoformans* is the closest *Bacillus* species according to 16S rRNA gene comparison. The ANIm values between strains ZYK^T^ and *B*. *azotoformans*, *B*. *subtilis subsp*. *subtilis* NCIB 3610^T^ were 84.4–84.7% and 85.4–86.7, respectively (Supplementary Tables S1 and S2). These values were below standard ANI criteria for species identity (95–96%).

### Selenite, nitrate reduction, and prediction metabolic pathway of dissimilatory nitrate reduction to ammonium

**Figure 1:**
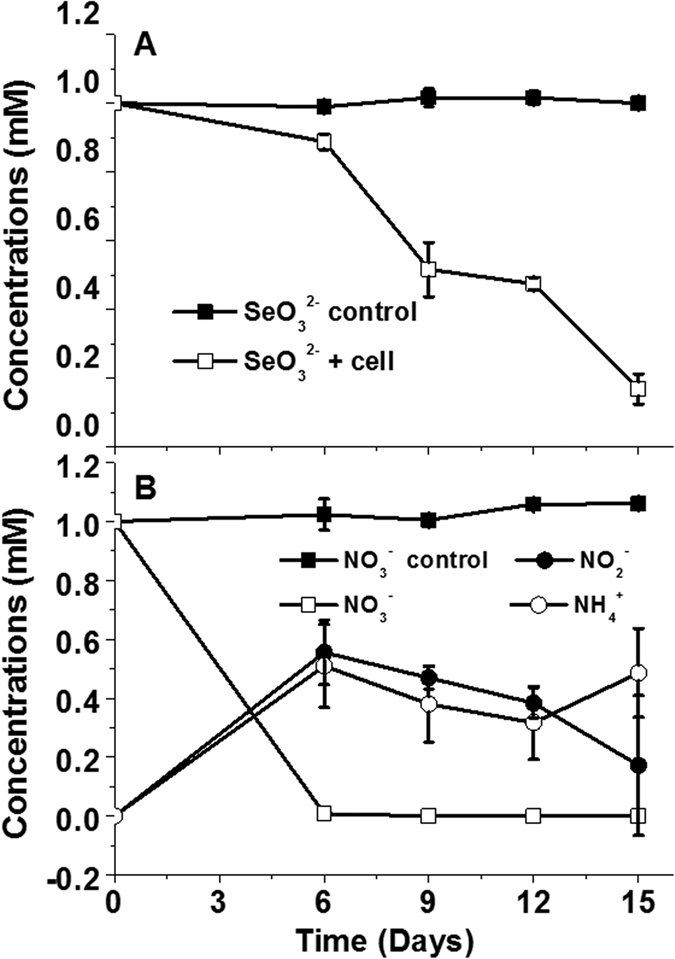
Selenite reduction (**A**) and nitrate reduction with nitrous oxide and ammonia generation (**B**) by strain ZYK^T^.

Strain ZYK^T^ reduced SeO_3_^2−^ (up to 80% of 1 mM over 15 days) with production of a red precipitate after 15 days of incubation (Fig. 1A). Figure 1B shows the reduction of NO_3_^−^ to nitrite and ammonium over 15 days of incubation.

**Figure 2:**
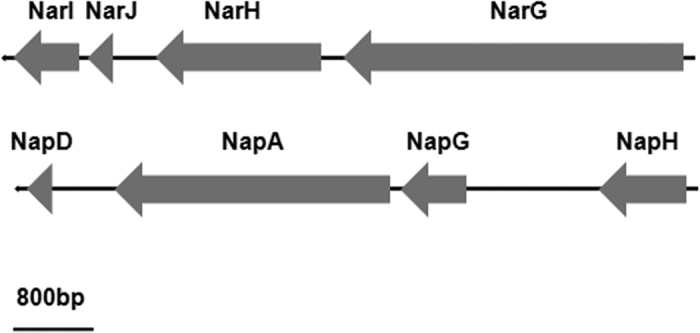
Operons encoding putative nitrate reductases of strain ZYK^T^.

**Figure 3:**
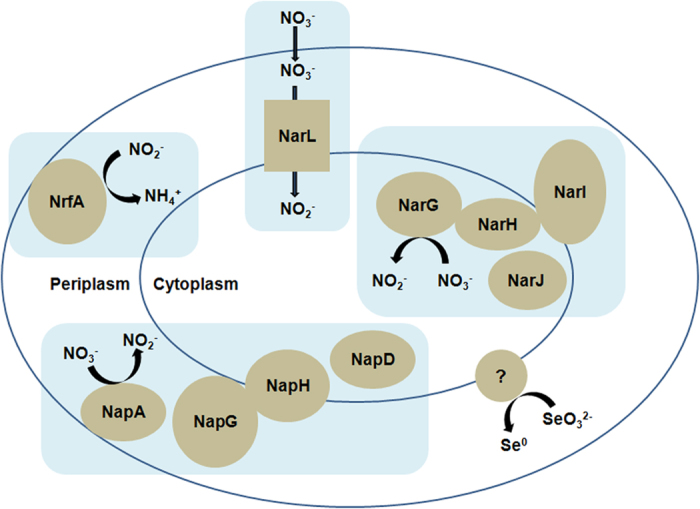
Proposed model of terminal dissimilatory nitrate, nitrite and selenite reductions in strain ZYK^T^. Proteins in blue block are encoded by the same operon. Uptake of molecule is represented by the direction of the arrows. Ovals: subunits of putative reductases; Circular: putative selenite reductase; Rectangles: putative transporters.

Genes encoding for proteins *Bacillus* ZYK-3GL000484, *Bacillus* ZYK-3GL000748, *Bacillus* ZYK-3GL000884 were identified in the genome of strain ZYK^T^ that may be responsible for the metabolism of nitrogen. The predicted protein sequence of NarG (*Bacillus* ZYK-3GL000484) in strain ZYK^T^ was 83% similar to NarG found in *B*. *licheniformis* 9945A. The respiratory nitrate reductase NarG subunit (1228 amino acids) forms a heterotrimetric structure with subunit NarH and NarI^22^,^29^ (Fig. 2). NarI has been shown to anchor the other subunits to the cytoplasmic membrane^29^. Thus, the presence of a NarI homolog in strain ZYK^T^ suggests that NarGH is oriented towards the cytoplasmic side of the membrane^22^,^30^ (Fig. 3). The respiratory nitrate reductase subunit NarJ plays an integral role in assembly of the NarGHI membrane complex^22^,^31^, and is predicted to be involved in the transport of nitrate into the cell. A transporter encoded by *narL* is predicted to transport nitrate into the cell or reduce nitrate into nitrite.

*Nap* operons have been identified in many prokaryotes, and Nap is found in the soluble periplasmic fraction^32^. In strain ZYK^T^, the predicted protein sequence of NapA (*Bacillus* ZYK-3GL000748) (850 amino acids long) was 83% similar to NapA found in *B*. *azotoformans* ATCC 29788^T^. The catalytic subunit encoded by *napA* is responsible for the reduction of nitrate to nitrite. Although the function of NapD is not understood, it was speculated to be necessary in maturation of NapA^33^. *napG* and *napH* are predicted to encode for a membrane-bound menaquinol complex^22^,^33^ (Figs 2 and 3).

Strain ZYK^T^ is capable of reducing nitrite to ammonium (Fig. 1B). We identified *nrfA*, which encodes for a membrane-localized protein, which is probably responsible for nitrite reduction in periplasm^22^. The predicted protein sequence of NrfA (*Bacillus* ZYK-3GL000884) (477 amino acids long) was 82% similar to that found in *Bacillus azotoformans* ATCC 29788^T^ (Fig. 3).

### Identification of SeNPs High-Affinity Proteins

We separated SeNPs high-affinity proteins from strain ZYK^T^ by SDS-PAGE (Fig. S5). Nano-LC-LTQ results showed a qualitative characterization of high-affinity proteins associated with self-assembled SeNPs in ZYK^T^ cells (Fig. S6). Proteins with imino groups tend to bind with elemental selenium through the formation of a Se-NH-bond, which leads to highly stable SeNPs structures^34^,^35^. Hence, proteins play a critical role in the nucleation and self-assembly of SeNPs^15^,^16^. The protein types with the highest number of peptides matched (46% of total) are hypothetical proteins because of insufficient information in the database (Table S3). About 27% of the high-affinity proteins were identified as energy metabolic enzymes, 12% as replication and transcription factors and 9% as amino acid metabolic enzymes.

### Inhibition of cancer cells H157 by SeNPs

**Figure 4:**
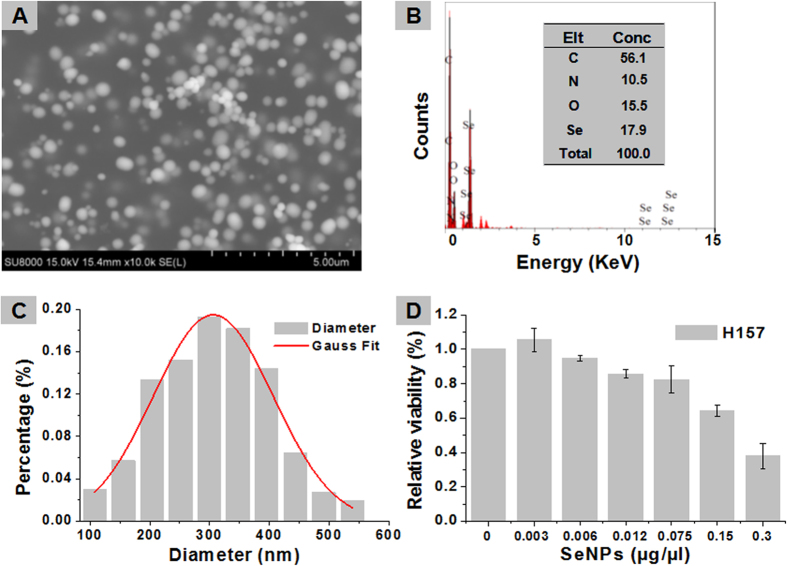
Representative SEM images of SeNPs from strain ZYK^T^ (**A**), The scale is 0.5 μm. Energy dispersive X-ray spectroscopy (EDX) spectrum of the electron-dense SeNPs (**B**). Corresponding size distribution histograms (**C**). Dose-dependent inhibitions of SeNPs on the viability of cancer cell line H157 following 24 h exposure (**D**).

Biogenic SeNPs of strain ZYK^T^ show a narrow size distribution and in line with the gaussian distribution (Fig. 4A–C). Dose-dependent effects of SeNPs on viability of H157 cells show that SeNPs are significantly toxic to H157 cells, only less than 40% cells survive expose to 0.3 μg/μl wet weight SeNPs (Fig. 4D). The results were superior to the effect of chemical generated SeNPs on HepG2 cells which the survival ratio is the same but with nearly 2.0 μg/μl SeNPs^35^.

## Discussion

In the absence of oxygen, many *Bacillus* species can respire on nitrate, but the molecular and genetic basis remains poorly characterized. The comparative genomic analysis of strain ZYK^T^ imply that it shares more orthologues with *B*. *subtilis subsp*. *subtilis* NCIB 3610^T^ than with *B*. *azotoformans*, although *B*. *azotoformans* is the closest *Bacillus* species according to 16S rRNA gene comparison. Three putative genes that encode for Nar, Nap and Nrf reductases were assigned through sequence comparison. The reductases were closely related to those of *B*. *azotoformans* and *B*. *licheniformis*. Their locations on either cytoplasmic or periplasmic side were also predicted. Strain ZYK^T^ carries multiple copies of key denitrification genes, encodes both membrane-bound and periplasmic nitrate reductases, and the key genes for nitrite reduction. Modularity and redundancy in nitrate reduction pathways may be a general feature of nitrate-reducing members of *Bacillus*. Unlike Gram-negative bacteria, Gram-positive microorganisms typically have a very limited space in their periplasm^36^,^37^. In this respect, most DNRA enzymes were consistently associated with membrane fractions in ZYK^T^. Considering the wide-spread occurrence of *Bacillus* species in paddy soil^5^, the *in situ* activity of *Bacillus*–mediated DNRA warrants further research.

We could not identify a selenite reductase gene because of the lack of reference sequences. Selenite reduction in ZYK^T^ may be performed by Nrf as suggested for *Thauera selenatis* and *Clostridium* sp. BXM^11^,^38^. Proteomic analysis of SeNPs high-affinity proteins shows abundant proteins with diverse cellular functions. As a general microbial detoxification reaction to oxyanions, selenite can be reduced to elemental Se by thiol groups of proteins/peptides such as peroxiredoxins and NADH peroxidase through “Painter-type” reaction^17^,^35^. In this study, no peroxiredoxins or NADH peroxidase was found in those high-affinity proteins, here the mechanism for selenite reduction is not clear. The energy metabolic enzymes may also relate to selenite reduction located in periplasm. The hypothetical proteins might be produced for intracellular binding with SeNPs and subsequent export of SeNPs. Further study is needed on the association of the protein binding with nanoparticles, and hypothetical proteins should be identified as well.

Microbial redox transformations of metals can result in the precipitation of metal nanoparticles across a wide range of environmental conditions^39^. The aggregation state of these particles may have a strong impact on metal mobility. Our results suggest that aggregation induced by elemental selenium high-affinity proteins plays an important role in limiting elemental selenium dispersal and thus influence the fate of selenium in paddy soils.

Here we demonstrate that the SeNPs generated by ZYK^T^ show a stronger inhibition effect on lung cancer cells than HepG2 cells and human breast carcinoma cells MDA-MB-231 treated by chemically generated SeNPs^35^,^40^. This protein derived control of biogenic SeNPs could have implications for industrial-scale production. SeNPs have also been tested *in vivo* with less toxicity compared with other seleno-compounds^41^. Further investigation on the underlying molecular mechanisms revealed that depletion of mitochondrial membrane potential and generation of superoxide anions contributed to SeNPs-induced apoptotic R-HepG2 cell death^41^. The size of SeNPs plays an important role in their biologic activity, and nanoparticle surface-induced oxidative stress has been confirmed as side effect, especially <100 nm, which means the smaller the better^42^. Nanoparticles of various sizes and chemical compositions preferentially mobilize to mitochondria, giving the possibility of ROS production and therefore interfering with antioxidant defenses^21^. Future study should be focus on reducing average diameter of biogenic SeNPs to improve their application in cancer therapy.

### Description of *Bacillus oryziterrae* sp. nov

*Bacillus oryziterrae* (oryziterrae: o.ry.zi.ter’rae. L. n. oryza, rice; L. n. terra, earth, soil; N. L. gen. n. oryziterrae, of the rice soil or field, referring to the source of the type strain). Cells are Gram-positive, spore-forming, motile, flagellated, bacilliform (0.2–0.3 μm wide and 1.5–2.0 μm long) rod. Facultative anaerobic. Grows optimally at pH 7.0 (range 6.0–7.6), 30 °C (range 21–45 °C) without NaCl (range 0–1.1%). Positive for nitrate and selenite reduction, and acid production from D-glucose and maltose. Negative for acid production from D-mannose, D-mannitol and D-xylose, and hydrolysis of casein, starch, gelatin and aesculin, can use citrate, L-histidine and tween-20 as carbon sources. The major cellular fatty acids are iso-C_14:0_ (17.8%), iso-C_15:0_ (17.8%), anteiso-C_15:0_ (10.4%), iso-C_16:0_ (10.5%) and C_16:0_ (32.0%). Polar lipid profile of strain contains PE, phosphatidylethanolamine; DPG, diphosphatidylglycerol; PG, phosphatidylglycerol; UPAL, unidentified aminophospholipid. The genomic DNA G+C content is 36.1 mol%.

The type strain is ZYK^T^ (=DSM 26460^T^ =CGMCC 1.5179^T^), isolated from a rice paddy soil sample, collected from Dehong, Yunnan, China.

## Methods

### Enrichment and isolation

A soil slurry for the enrichment of anaerobic bacteria was established from a rice paddy soil sample, collected from Dehong, Yunnan (24°41′29″N, 97°58′37″E), China. The vessels with soil slurry were fully filled, sealed with a stopper and kept at 4 °C until used for culture setup. To determine soil characteristics the soil was air-dried, ground with a mortar and crushed to pass through a 2.0 mm sieve. Soil organic carbon (2.3%) was determined by potassium dichromate oxidation titration, and total Fe (28.4 g kg^−1^) was determined by inductively coupled plasma optical emission spectroscopy (ICP-OES). Plant-available sulfur (14.4 mg kg^−1^) was extracted by Ca(H_2_PO_4_)_2_ (0.01 mol l^−1^) and determined by turbidimetry. Soil-available Fe (36.5 mg kg^−1^) was extracted by DTPA-CaCl_2_-TEA and determined by ICP-OES. Soil-available Mn (17.1 mg kg^−1^) was extracted by ammonium acetate (1.0 mol l^−1^) and determined by ICP-OES.

An anoxic freshwater medium was used for cultivation^22^. To establish anoxic cultures, fresh soil sample (5.0 g) was mixed with 100 ml anoxic water, then 5.0 ml of soil suspension was added to a serum bottle containing 50 ml medium and incubated at 30 °C in the dark. Anaerobic culturing techniques were used throughout the enrichment^43^. During incubation, the enrichment culture was briefly shaken once a day. After 7 days growth, new cultures were inoculated (10% v/v), and after 15 transfers, a stable culture was obtained. For the isolation of bacteria, the method was the dilution to extinction technique. The final dilution culture was purified by streaking on agar plates containing sodium selenite in anoxic jars under an atmosphere of 15% O_2_ and 6% CO_2_. The single colony that produced red precipitate was chosen, because selenite reduction products, SeNPs is in red color.

### Characterization of strain ZYK^T^

The cell morphology and spore formation of strain ZYK^T^ was assessed by phase-contrast light microscopy (Nikon E-2100). The 16S rRNA gene sequence (1,544 nt) of strain ZYK^T^ was used to identify related taxa using the BLAST program^22^,^44^ and EzTaxon server 2.1 (http://eztaxon-e.ezbiocloud.net). The 16S rRNA gene sequences of related taxa were obtained from GenBank. In total, 299 *Bacillus* sequences were retrieved from the database and CLUSTAL 2.0^19^,^45^ used to align the sequences. A phylogenetic tree was constructed using the maximum likelihood method in MEGA 5.05 with bootstrap analysis based on 1000 replications^22^,^46^,^47^.

Experiments comparing strain ZYK^T^^22^ to other *Bacillus* species were conducted in duplicate to test pH, temperature and NaCl ranges for growth in anoxic freshwater medium. Strains ZYK^T^, *Bacillus azotoformans* ATCC 29788^T^, *Bacillus cohnii* DSM 6307^T^ and *Bacillus subtilis* subsp. *subtilis* NCIB 3610^T^ were sub-cultured at least once under the same experimental conditions. For pH studies, the medium was adjusted with anaerobic stock solutions of either HCl or NaOH to give the desired pH of 6.0, 6.2, 6.6, 7.0, 7.3, 7.6, or 7.8. Temperature range for growth was determined at 21, 25, 30, 37, 40, and 45 °C. NaCl requirement/tolerance was tested at concentrations of 0, 0.2, 0.4, 0.6, 0.8, and 1.1% (w/v). The reduction of nitrate and selenite was tested at a concentration of 1.0 mM and monitored by ion chromatography (GP50 gradient pump, a column oven LC25, an electrochemical detector ED50) with a Dionex Ionpac AS14 column (4.6 × 3100 mm). Catalase activity was tested by adding 3% H_2_O_2_ to culture plates. The oxidase reaction was performed on filter paper moistened with a 1% (w/v) aqueous solution of N, N, N′, N′-tetramethyl-*p*-henylenediamine. Carbon source utilization was tested with sodium citrate, D-cellobiose, L-arginine, L-proline, L-serine, L-histidine, and Tween-20 in the medium. Hydrolysis of casein, starch, gelatin and aesculin was determined according to the modified methods described by Lanyi^48^ and Smibert & Krieg^49^. Acid production from D-mannose, D-glucose, maltose, D-mannitol and D-xylose was tested as described^50^.

The bacterial culture grown at 30 °C for 7 days was used for lipid extraction according to a procedure^51^ employing a single-phase organic solvent system comprised of sodium hydroxide, methanol, distilled water, concentrated hydrochloric acid, n-hexane, methyl tertiary butyl ether. After extraction, 3 ml sodium hydroxide and a few drops of saturated sodium chloride solution were added to the organic-phase, which resulted in a two-phase system. The lipids confined to the upper phase were collected. The lipids were identified by the Sherlock Microbial Identification System (MIDI) using an Agilent 6890 series gas chromatograph (GC-FID) equipped with a 25 m UItra-2 column (0.22-mm internal diameter, 0.33 μm film thickness). Polar lipids of strain ZYK^T^ and reference strain *Bacillus azotoformans* ATCC 29788^T^ were extracted and analyzed by two-dimensional TLC^52^.

### Genome synteny comparisons

Pairwise alignments for dot plot representations were performed on the six-frame amino acid translation of the genome sequences of strain ZYK^T^ (ANOK00000000) *Bacillus azotoformans* ATCC 29788^T^ (AJLR00000000) and *Bacillus subtilis* subsp. *subtilis* NCIB 3610^T^ (ABQL01000000) using the Promer program in the MUMmer 3.23 package^53^. For all analyses default parameters were applied; that is, exact matches longer than six amino acids were identified, adjacent exact matches were joined if separated by a gap no longer than 30 amino acids, the resulting clusters were further processed if the total length of their matches was longer than 20 amino acids and then aligned using a BLOSUM62 amino acid substitution matrix. The average nucleotide identity (ANI) estimate values between two genomes were calculated using JSpecies (V1.2.1).

### Selenite and nitrate reduction analysis

To examine the reduction of selenite and nitrate strain ZYK^T^ was cultivated anaerobically with either selenite (1.0 mmol l^−1^) or NaNO_3_ (1.0 mmol l^−1^) in 100 ml serum bottles in the dark. Each batch of experiments were established in triplicate, inoculated with 1.0 ml (OD_600_ 0.16) of an exponential phase culture, and incubated at 30 °C, respectively. Samples were taken from the medium after 0, 6, 9, 12, and 15 days of incubation and analyzed for nitrate, nitrite and ammonium. To determine nitrate and nitrite, 0.5 ml of the sample was filtered (0.22 μm) in order to remove particulates that could interfere with ion chromatography. The ion chromatography system consisted of a GP50 gradient pump (Thermo Fisher Scientific Inc. Sunnyvale, California, USA), a column oven LC25, an electrochemical detector ED50 and a Dionex Ionpac AS14 column (4.6 × 3100 mm). The operating condition was operated as described^11^, with an eluent of 3.5 mmol l^−1^ Na_2_CO_3_ and 1.0 mmol l^−1^ NaHCO_3_ at a flow rate of 1.2 ml min^−1^. The measurement of ammonium was performed by the indophenol-blue colorimetric method and the concentration of ammonium monitored by spectrophotometry (Pgeneral T6, Beijing, China). The measurement of selenite reduction was performed by ion chromatography, and the selenite reduction product, elemental selenium particles, was identified by field emission scanning electron microscopy (FESEM, HITACHI SU8020).

### Phylogenetic analysis of nitrate reductases

The protein sequence of putative nitrate reductases Nar, Nap and Nrf, as well as selected putative homologues of the catalytic subunits retrieved via BLASTP searches^45^ of non-redundant databases at NCBI, were aligned using CLUSTALW^54^ and analyzed in MEGA 5.05^46^.

### Proteome analysis of SeNPs high-affinity proteins: protein identification, quantification, database construction, and bioinformatic data analysis

SeNPs were extracted from ZYK^T^ culture grown with 1.0 mM selenite for 15 days. SeNPs were treated in loading buffer to dissolve high-affinity proteins, and 20 μl suspension was loaded onto sodium dodecyl sulfate-polyacrylamide gel electrophoresis (SDS-PAGE). The samples were then loaded onto an Agilent C_18_ trap, followed by nano-LC-ESI-MS/MS analysis^55^. The peptides were sequentially eluted from the HPLC column with a gradient of 0–90% of buffer B (acetonitrile: water: acetic acid, 80: 19.9: 0.1) in buffer A (acetonitrile: water: acetic acid, 5: 94.9: 0.1) at a flow rate of approximately 0.5 ml min^−1^ (after split) using surveyor pumps. The eluted peptides were sprayed directly from the tip of the capillary column to the LTQ mass spectrometer (Thermo Finnigan, San Jose, CA) for mass spectrometry analysis. The LTQ mass spectrometer was operated in the data-dependent mode in which first the initial MS scan recorded the mass to charge (m/z) ratios of ions over the mass range from 300–1800 Da. The five most abundant ions were automatically selected for subsequent collision-activated dissociation.

All MS/MS data were searched against the predictive protein sequence according to the genomic data of strain ZYK^T^ (ANOK00000000) and the NCBI database using the SEQUEST (v.28). All searches were performed using a precursor mass tolerance of 3.0 Da calculated using average isotopic masses. Variable modification was set for methionine with the addition of 15.999 Da to represent methionine oxidation, static modification was set for cysteine with the addition of 57.052 Da to represent cysteine carboxyamidation; a fragment ion mass tolerance of 1.0 Da was used. Enzyme cleavage specificity was set to trypsin and no more than two missed cleavages were allowed. The SEQUEST outputs were analyzed using commercial software Thermo Electron BioWorks (Rev.3.3.1 sp1). The filter settings for peptides were as follows:- distinct peptide, Xcorr ≥ 1.9 (z = 1), 2.7 (z = 2), 3.50 (z = 3), Sp ≥ 500, Rsp ≤ 5, at least two distinct peptides per protein.

### Cancer cell viability measurements

To test for the toxicity of SeNPs on cancer cells, a common and fatal cancer, non–small lung cancer cell line H157 was chosen. Cell line H157 bought from the Cell Culture Center of the Institute of Basic Medical Sciences, Chinese Academy of Medical Sciences School of Basic Medicine Peking Union Medical College. H157 cells were grown in RPMI medium with 10% FBS and 1% PEST at 37 °C in a 5% CO_2_ incubator^55^. Viability was measured by means of the MTT viability kit in 96-well cell culture plates (Nunc). Cells were counted in a Burke’s chamber, and 10,000 cells per well (200,000 cells/ml) were plated and pre-incubated for 24 h to allow adhesion. After pre-incubation, the medium was discarded, and treated with a series of dosages of SeNPs (0.003, 0.006, 0.012, 0.075, 0.15, and 0.3 μg μl^−1^ wet weight, in 50 μl RPMI 1640) for 24 h in five replicates. Determination of cells viability was performed according to the operating instructions.

### Nucleotide sequence accession numbers

The GenBank accession number for the 16S rRNA gene sequence of strain ZYK^T^ is JX103165. A draft genome is available under accession number ANOK00000000.

## Additional Information

**How to cite this article**: Bao, P. *et al*. Characterization and Potential Applications of a Selenium Nanoparticle Producing and Nitrate Reducing *Bacterium Bacillus* oryziterrae sp. nov. *Sci. Rep.* **6**, 34054; doi: 10.1038/srep34054 (2016).

## Supplementary Material

Supplementary Information

## Figures and Tables

**Table 1:** Phenotypic characteristics that differentiate strain ZYK^T^ from related *Bacillus* species^25^,^26^.

Characteristic	1	2	3	4
Cell size	0.2–0.3 × 1.0–2.0	1.0 × 2–3	0.6–0.7 × 2–3	0.5 × 2–3
Gram	+	+	+	+
Motility	+	+	+	+
Flagellation	+	+	+	+
Spore shape	O	O	O	C
pH range	6.0–7.6	5.6–8.4	4.5–9.9	5.5–8.6
Optimum pH	7.0–7.2	6.8	8.7–9.2	6.8
NaCl (%) range	0–1.1	0–5	0–5	0–7
Colony	white	white	Cream white	Cream white
Temperature (°C) range	21–45	4–46	10–47	15–55
Temperature (°C) optimum	30	30	37	28–30
Oxygen requirements	Facultatively anaerobic	Facultatively anaerobic	Strictly aerobic	Strictly aerobic
Catalase	−	−	+	+
Oxidase	−	+	+	−
Nitrate reduction	+	+	+	+
Selenite reduction	+	−	+	−
Acid production from:				
D-Mannose	−	+	−	+
D-Glucose	+	−	−	+
D-Mannitol	−	−	−	+
D-xylose	−	−	−	+
Maltose	+	−	−	+
Hydrolysis of:				
Aesculin	−	−	−	−
Casein	−	+	+	+
Starch	−	−	+	+
Gelatin Utilization of:	−	−	+	+
Citrate	+	+	+	+
D-Cellobiose	−	−	−	−
L-Arginine	+	−	−	+
L-Proline	−	+	+	−
L-Serine	−	−	+	−
L-Histidine	+	−	−	−
Tween-20	+	+	+	+
DNA G+C content (mol %)	36.1	45.0	36.9	50.3

Strains: 1, ZYK^T^ (present study); 2, *Bacillus azotoformans* ATCC 29788^T^; 3, *Bacillus cohnii* DSM 6307^T^; 4, *Bacillus subtilissubsp*. *subtilis* NCIB 3610^T^. O, Oval; C, Cylindrical; +, Positive; −, Negative.

**Table 2:** Fatty acid composition (%) of strain ZYK^T^ and related *Bacillus azotoformans*.

Fatty acid	1	2	3
C_9:0_	1.9	—	—
iso-C_14:0_	17.8	10.6	—
C_14:0_	4.7	3.1	—
iso-C_15:0_	17.8	17.1	—
anteiso-C_15:0_	10.4	37.9	42.2
C_15:0_	—	2.5	—
iso-C_16:0_	10.5	4.4	—
C_16:0_	32	15.6	28.2
anteiso-C_17:0_	—	2.5	29.7
C_18:0_	4.9	1.3	—

Strains: 1, Strain ZYK^T^ (present study); 2, *Bacillus azotoformans* ATCC 29788^T^; 3, *Bacillus cohnii* DSM 6307^T^.
