# Supplementary material for: Characterization and Potential Applications of a Selenium Nanoparticle Producing and Nitrate Reducing Bacterium Bacillus oryziterrae sp. nov
Source: Sci Rep. 2016 Sep 28;6:34054. doi: 10.1038/srep34054 (PMC5039721; doi:10.1038/srep34054)
Supplement: Supplementary Information [file srep34054-s1.pdf]

Table S1. ANIm values for strain ZYK^T, *Bacillus azotoformans* NBRC 15712^T and *Bacillus subtilis* subsp. *subtilis* NCIB 3610^T.

Strains	1	2	3
1 ZYK^T	-	84.38	85.37
2 *Bacillus azotoformans* NBRC 15712^T	84.71	-	84.63
3 *Bacillus subtilis* subsp. *subtilis* NCIB 3610^T	86.73	84.62	-

Table S2. Tetranucleotide regression values for strain ZYK^T, *Bacillus azotoformans* NBRC 15712^T and *Bacillus subtilis* subsp. *subtilis* NCIB 3610^T.

Strains	1	2	3
1 ZYK^T	-	0.95542	0.71906
2 *Bacillus azotoformans* NBRC 15712^T	0.95542	-	0.78896
3 *Bacillus subtilis* subsp. *subtilis* NCIB 3610^T	0.71906	0.78896	-

Table S3. Proteomics analysis: identification of SeNPs high-affinity proteins.

Function description	Score	Molecule weight	Accession number	Peptides (Hits)
hypothetical protein				
hypothetical protein [Bacillus sp. ZYK]	280.32	84582.1	516366525	28
hypothetical protein [Bacillus sp. ZYK]	190.28	88911.9	516366029	19
hypothetical protein [Bacillus sp. ZYK]	180.27	71259.9	516366425	18
hypothetical protein [Bacillus sp. ZYK]	140.29	99804.0	516366048	14
hypothetical protein [Bacillus sp. ZYK]	140.25	82608.7	516363766	14
hypothetical protein [Bacillus sp. ZYK]	120.27	47397.9	516363478	12
hypothetical protein [Bacillus sp. ZYK]	120.26	71877.2	516363492	12
hypothetical protein [Bacillus sp. ZYK]	120.26	102357.4	516366440	12
hypothetical protein [Bacillus sp. ZYK]	110.32	50500.0	516365678	11
hypothetical protein [Bacillus sp. ZYK]	110.32	35549.1	516365798	11
hypothetical protein [Bacillus sp. ZYK]	98.31	48318.9	516365964	10
hypothetical protein [Bacillus sp. ZYK]	90.27	37121.5	516363477	9
hypothetical protein [Bacillus sp. ZYK]	80.30	36183.4	516366663	8
hypothetical protein [Bacillus sp. ZYK]	80.28	33988.1	516363495	8
hypothetical protein [Bacillus sp. ZYK]	80.28	48024.5	516366290	8
hypothetical protein [Bacillus sp. ZYK]	80.22	52884.1	516363845	8
hypothetical protein [Bacillus sp. ZYK]	70.31	44658.8	516366446	7
hypothetical protein [Bacillus sp. ZYK]	70.24	36360.1	516363511	7
hypothetical protein [Bacillus sp. ZYK]	60.32	51688.2	516366506	6
hypothetical protein [Bacillus sp. ZYK]	60.31	40528.9	516365719	6
hypothetical protein [Bacillus sp. ZYK]	60.29	46125.6	516363605	6
hypothetical protein [Bacillus sp. ZYK]	60.26	32765.6	516363935	6
hypothetical protein [Bacillus sp. ZYK]	60.25	43333.8	516363507	6
hypothetical protein [Bacillus sp. ZYK]	60.25	49177.4	516363790	6
hypothetical protein [Bacillus sp. ZYK]	60.25	82658.6	516365892	6
hypothetical protein [Bacillus sp. ZYK]	60.24	39723.8	516364243	6
hypothetical protein [Bacillus sp. ZYK]	60.23	50235.0	516366735	6
hypothetical protein [Bacillus sp. ZYK]	60.21	40581.3	516366763	6
hypothetical protein [Bacillus sp. ZYK]	58.21	48464.0	516365612	6
hypothetical protein [Bacillus sp. ZYK]	50.31	37993.0	516365653	5
hypothetical protein [Bacillus sp. ZYK]	50.29	75023.2	516364478	5
hypothetical protein [Bacillus sp. ZYK]	50.29	34224.5	516365956	5
hypothetical protein [Bacillus sp. ZYK]	50.26	28155.0	516366172	5
hypothetical protein [Bacillus sp. ZYK]	50.26	54318.9	516366258	5
hypothetical protein [Bacillus sp. ZYK]	50.25	22954.4	516366721	5
hypothetical protein [Bacillus sp. ZYK]	50.25	30939.9	516365938	5
hypothetical protein [Bacillus sp. ZYK]	50.24	49383.0	516364885	5
hypothetical protein [Bacillus sp. ZYK]	50.24	79926.9	516366115	5
hypothetical protein [Bacillus sp. ZYK]	50.23	65348.9	516364182	5

hypothetical protein [Bacillus sp. ZYK]	50.23	61086.1	516363780	5	
hypothetical protein [Bacillus sp. ZYK]	50.23	56056.9	516364772	5	
hypothetical protein [Bacillus sp. ZYK]	50.20	39263.4	516365706	5	
hypothetical protein [Bacillus sp. ZYK]	48.28	55712.5	516363378	5	
hypothetical protein [Bacillus sp. ZYK]	48.25	75749.2	516363450	5	
hypothetical protein [Bacillus sp. ZYK]	48.18	34985.9	516366472	5	
hypothetical protein [Bacillus sp. ZYK]	40.31	60859.1	516365935	4	
hypothetical protein [Bacillus sp. ZYK]	40.28	41869.1	516366365	4	
hypothetical protein [Bacillus sp. ZYK]	40.28	62244.4	516365595	4	
hypothetical protein [Bacillus sp. ZYK]	40.28	27826.9	516366019	4	
hypothetical protein [Bacillus sp. ZYK]	40.27	35696.2	516366067	4	
hypothetical protein [Bacillus sp. ZYK]	40.24	113433.5	516366445	4	
hypothetical protein [Bacillus sp. ZYK]	40.23	33276.4	516365983	4	
hypothetical protein [Bacillus sp. ZYK]	40.23	91785.8	516366471	4	
hypothetical protein [Bacillus sp. ZYK]	40.23	50356.1	516365776	4	
hypothetical protein [Bacillus sp. ZYK]	40.22	51386.7	516365061	4	
hypothetical protein [Bacillus sp. ZYK]	40.22	73473.7	516365351	4	
hypothetical protein [Bacillus sp. ZYK]	40.22	136129.0	516363663	4	
hypothetical protein [Bacillus sp. ZYK]	40.21	72006.6	516364647	4	
hypothetical protein [Bacillus sp. ZYK]	40.19	55320.6	516366474	4	
hypothetical protein [Bacillus sp. ZYK]	40.19	51920.3	516365204	4	
hypothetical protein [Bacillus sp. ZYK]	40.18	34764.5	516363794	4	
hypothetical protein [Bacillus sp. ZYK]	38.28	20242.4	516366566	4	
hypothetical protein [Bacillus sp. ZYK]	38.24	46125.2	516364485	4	
hypothetical protein [Bacillus sp. ZYK]	38.21	77847.3	516364038	4	
hypothetical protein [Bacillus sp. ZYK]	30.30	18308.8	516365533	3	
hypothetical protein [Bacillus sp. ZYK]	30.26	32764.5	516366180	3	
hypothetical protein [Bacillus sp. ZYK]	30.25	44983.4	516365221	3	
hypothetical protein [Bacillus sp. ZYK]	30.25	45355.6	516363482	3	
hypothetical protein [Bacillus sp. ZYK]	30.25	45980.5	516363623	3	
hypothetical protein [Bacillus sp. ZYK]	30.24	74148.1	516364635	3	
hypothetical protein [Bacillus sp. ZYK]	30.24	57303.4	516365588	3	
hypothetical protein [Bacillus sp. ZYK]	30.24	52131.4	516365356	3	
hypothetical protein [Bacillus sp. ZYK]	30.22	40481.1	516365540	3	
hypothetical protein [Bacillus sp. ZYK]	30.22	17450.4	516363743	3	
hypothetical protein [Bacillus sp. ZYK]	30.22	42433.4	516366647	3	
hypothetical protein [Bacillus sp. ZYK]	30.20	31045.9	516365691	3	
hypothetical protein [Bacillus sp. ZYK]	30.20	38461.2	516365343	3	
hypothetical protein [Bacillus sp. ZYK]	30.19	41618.1	516364950	3	
hypothetical protein [Bacillus sp. ZYK]	30.19	49248.9	516363789	3	
hypothetical protein [Bacillus sp. ZYK]	30.18	67027.5	516365592	3	
hypothetical protein [Bacillus sp. ZYK]	30.18	55911.6	516365110	3	
hypothetical protein [Bacillus sp. ZYK]	28.25	72923.7	516366097	3	
hypothetical protein [Bacillus sp. ZYK]	20.29	37906.4	516365751	2	

hypothetical protein [Bacillus sp. ZYK]	20.27	126295.0	516366064	2	
hypothetical protein [Bacillus sp. ZYK]	20.27	65853.2	516366168	2	
hypothetical protein [Bacillus sp. ZYK]	20.26	73068.9	516366436	2	
hypothetical protein [Bacillus sp. ZYK]	20.26	60704.9	516363655	2	
hypothetical protein [Bacillus sp. ZYK]	20.26	32433.4	516366715	2	
hypothetical protein [Bacillus sp. ZYK]	20.26	23126.6	516365902	2	
hypothetical protein [Bacillus sp. ZYK]	20.26	33860.7	516363639	2	
hypothetical protein [Bacillus sp. ZYK]	20.25	30230.8	516364123	2	
hypothetical protein [Bacillus sp. ZYK]	20.25	49285.6	516366069	2	
hypothetical protein [Bacillus sp. ZYK]	20.25	30139.0	648400677	2	
hypothetical protein [Bacillus sp. ZYK]	20.25	39866.2	516365658	2	
hypothetical protein [Bacillus sp. ZYK]	20.24	32888.6	516366269	2	
hypothetical protein [Bacillus sp. ZYK]	20.24	16540.6	516363863	2	
hypothetical protein [Bacillus sp. ZYK]	20.24	71393.1	516365679	2	
hypothetical protein [Bacillus sp. ZYK]	20.24	48091.6	516365671	2	
hypothetical protein [Bacillus sp. ZYK]	20.24	45081.2	516363731	2	
hypothetical protein [Bacillus sp. ZYK]	20.23	36220.3	516366519	2	
hypothetical protein [Bacillus sp. ZYK]	20.23	34805.8	516364464	2	
hypothetical protein [Bacillus sp. ZYK]	20.23	30528.9	516365785	2	
hypothetical protein [Bacillus sp. ZYK]	20.22	81478.9	516364786	2	
hypothetical protein [Bacillus sp. ZYK]	20.22	26660.7	516364574	2	
hypothetical protein [Bacillus sp. ZYK]	20.22	42910.6	516366405	2	
hypothetical protein [Bacillus sp. ZYK]	20.22	92113.7	516366174	2	
hypothetical protein [Bacillus sp. ZYK]	20.22	34095.9	516365579	2	
hypothetical protein [Bacillus sp. ZYK]	20.22	48276.8	516363811	2	
hypothetical protein [Bacillus sp. ZYK]	20.22	41252.4	516364856	2	
hypothetical protein [Bacillus sp. ZYK]	20.22	45821.0	516366090	2	
hypothetical protein [Bacillus sp. ZYK]	20.22	33381.7	516363727	2	
hypothetical protein [Bacillus sp. ZYK]	20.21	21447.3	516365143	2	
hypothetical protein [Bacillus sp. ZYK]	20.21	123845.1	516364651	2	
hypothetical protein [Bacillus sp. ZYK]	20.21	30907.5	516366111	2	
hypothetical protein [Bacillus sp. ZYK]	20.21	33631.6	516363659	2	
hypothetical protein [Bacillus sp. ZYK]	20.21	37211.5	516366382	2	
hypothetical protein [Bacillus sp. ZYK]	20.21	26325.7	516364126	2	
hypothetical protein [Bacillus sp. ZYK]	20.21	37728.6	516366196	2	
hypothetical protein [Bacillus sp. ZYK]	20.21	32998.3	516364310	2	
hypothetical protein [Bacillus sp. ZYK]	20.20	69172.2	516366518	2	
hypothetical protein [Bacillus sp. ZYK]	20.20	62576.4	516366005	2	
hypothetical protein [Bacillus sp. ZYK]	20.20	52265.4	516366169	2	
hypothetical protein [Bacillus sp. ZYK]	20.20	66885.4	516363895	2	
hypothetical protein [Bacillus sp. ZYK]	20.20	95354.2	516364215	2	
hypothetical protein [Bacillus sp. ZYK]	20.19	65396.3	516365683	2	
hypothetical protein [Bacillus sp. ZYK]	20.19	70717.6	516365220	2	
hypothetical protein [Bacillus sp. ZYK]	20.19	74355.1	516364563	2	

hypothetical protein [Bacillus sp. ZYK]	20.19	27745.0	516363648	2	
hypothetical protein [Bacillus sp. ZYK]	20.19	88714.9	516365389	2	
hypothetical protein [Bacillus sp. ZYK]	20.19	42236.8	516363758	2	
hypothetical protein [Bacillus sp. ZYK]	20.18	62002.8	516364768	2	
hypothetical protein [Bacillus sp. ZYK]	20.18	18424.5	516364199	2	
hypothetical protein [Bacillus sp. ZYK]	20.18	51173.8	516366624	2	
hypothetical protein [Bacillus sp. ZYK]	20.18	46188.7	516366009	2	
hypothetical protein [Bacillus sp. ZYK]	20.18	48360.8	516365860	2	
hypothetical protein [Bacillus sp. ZYK]	20.18	52365.3	516363849	2	
hypothetical protein [Bacillus sp. ZYK]	20.17	144066.6	516364505	2	
hypothetical protein [Bacillus sp. ZYK]	20.17	62650.2	516365972	2	
hypothetical protein [Bacillus sp. ZYK]	20.17	109904.9	516365008	2	
hypothetical protein [Bacillus sp. ZYK]	20.16	42328.6	516366496	2	
hypothetical protein [Bacillus sp. ZYK]	20.16	140734.1	516365410	2	
hypothetical protein [Bacillus sp. ZYK]	20.16	16677.0	516366073	2	
hypothetical protein [Bacillus sp. ZYK]	18.18	31341.9	516366422	2	
Energy metabolic enzymes [Bacillus sp. ZYK]					
glyceraldehyde-3-phosphate dehydrogenase	78.24	38047.4	516366045	8	
glyceraldehyde-3-phosphate dehydrogenase	60.23	36276.2	516366336	6	
fructose 1,6-bisphosphatase	20.22	33788.7	516366593	2	
fumarate hydratase	70.25	56103.5	516364368	7	
1-deoxy-D-xylulose-5-phosphate synthase	20.20	69839.9	516365686	2	
1-pyrroline-5-carboxylate dehydrogenase	150.29	56257.5	516364162	15	
2-isopropylmalate synthase	40.20	56531.8	516365969	4	
2-oxoacid ferredoxin oxidoreductase subunit beta	90.26	31518.5	516363802	9	
2-oxoglutarate ferredoxin oxidoreductase subunit alpha	208.28	63137.7	516363801	21	
2-oxoisovalerate dehydrogenase subunit alpha	50.23	36495.0	516365673	5	
2-oxoisovalerate dehydrogenase subunit beta	40.20	35994.3	516365672	4	
3-deoxy-7-phosphoheptulonate synthase	20.20	39665.4	516366114	2	
3-ketoacyl-ACP reductase	30.25	26163.9	516363660	3	
3-oxoacyl-ACP synthase	110.34	44080.7	516364594	11	
3-oxoacyl-ACP synthase	98.27	33358.0	516364593	10	
3-phosphoglycerate dehydrogenase	30.22	43443.3	516364437	3	
4-hydroxy-3-methylbut-2-en-1-yl diphosphate synthase	40.30	39016.9	516365772	4	
acetyl-CoA acetyltransferase	40.23	41898.0	516365639	4	
acetyl-CoA acetyltransferase	30.21	40843.5	516366606	3	
acetyl-CoA acetyltransferase	20.22	41091.8	516365132	2	
acetyl-CoA carboxylase biotin carboxylase subunit	80.29	50206.2	516365694	8	
acetyl-CoA hydrolase	30.23	55491.8	516364415	3	
acetyl-CoA synthetase	170.29	64537.2	516364506	17	
acetyl-CoA synthetase	90.22	64751.5	516366107	9	
aconitate hydratase	280.37	98275.7	516363858	28	
acyl-CoA dehydrogenase	60.32	41489.1	516366602	6	
acyl-CoA dehydrogenase	50.27	66819.4	648400785	5	

acyl-CoA dehydrogenase	20.26	41359.0	516366603	2
acyl--CoA ligase	110.33	58592.2	516366088	11
adenylate kinase	20.22	24053.4	516364810	2
adenylosuccinate synthetase	50.28	47491.7	516366701	5
aldehyde ferredoxin oxidoreductase	50.26	65551.9	516365166	5
citrate synthase	80.30	41595.5	516366055	8
CoA-binding protein	30.21	15541.9	516363896	3
catabolite control protein A	20.23	37175.4	516366113	2
enolase	98.29	46144.8	516366332	10
ATP metabolic enzymes [Bacillus sp. ZYK]				
ABC transporter ATP-binding protein	20.22	27838.1	516364245	2
ABC transporter substrate-binding protein	60.27	45775.5	516363941	6
ABC transporter substrate-binding protein	20.26	35070.7	648400642	2
C4-dicarboxylate ABC transporter	30.27	70482.9	516365253	3
excinuclease ABC subunit A	80.23	107115.8	516366368	8
excinuclease ABC subunit B	56.29	75677.9	516366369	6
F0F1 ATP synthase subunit beta	170.28	51474.0	516366563	17
F0F1 ATP synthase subunit epsilon	20.31	14281.5	516366562	2
F0F1 ATP synthase subunit gamma	20.17	31912.4	516366564	2
FAD-dependent pyridine nucleotide-disulfide oxidoreductase	20.26	19682.5	516365637	2
GMP synthase	40.20	57893.9	516364968	4
GTPase Era	20.26	34500.6	516365789	2
GTP-binding protein	60.21	68869.4	516363543	6
GTP-binding protein Der	30.26	48952.8	516365562	3
GTP-binding protein LepA	50.24	68055.9	516365812	5
GTP-binding protein YchF	30.26	40365.1	516366709	3
GTP-binding protein YsxC	20.18	21964.3	516365960	2
Clp protease ATPase	40.25	52649.2	648400602	4
heme ABC transporter ATP-binding protein	60.31	60788.1	516365149	6
AMP-dependent synthetase	20.16	59980.1	516364515	2
CTP synthetase	50.26	59906.9	516366598	5
glmZ(sRNA)-inactivating NTPase	20.16	33566.7	516366344	2
Electron transfer proteins [Bacillus sp. ZYK]				
cytochrome B6	30.24	25442.0	516365532	3
cytochrome C	20.27	61131.4	648400714	2
cytochrome C oxidase	30.26	69201.3	516363556	3
cytochrome CBB3	28.20	27743.7	516365531	3
electron transfer flavoprotein subunit alpha	80.32	33892.6	516366317	8
electron transfer flavoprotein subunit alpha	80.32	34076.8	516366018	8
electron transfer flavoprotein subunit alpha, partial	80.32	33203.8	648400692	8
electron transfer flavoprotein subunit beta	60.31	27991.5	516364982	6
ferredoxin--NADP reductase	90.25	36272.3	516366263	9
Replication and transcription proteins [Bacillus sp. ZYK]				

30S ribosomal protein S1	70.26	42154.8	516365568	7	
30S ribosomal protein S12 methylthiotransferase	30.27	51529.4	516365804	3	
30S ribosomal protein S2	30.23	26588.6	516363752	3	
30S ribosomal protein S3	40.27	24655.1	516364824	4	
50S ribosomal protein L1	40.35	26634.7	648400681	4	
50S ribosomal protein L13	20.18	16503.9	516364798	2	
50S ribosomal protein L14	30.19	13221.4	516364821	3	
50S ribosomal protein L19	20.17	13248.5	516363672	2	
50S ribosomal protein L21	20.17	11241.1	516365916	2	
50S ribosomal protein L3	20.29	22915.2	516364830	2	
50S ribosomal protein L7/L12	20.18	12730.6	516364841	2	
DNA topoisomerase IV subunit A	80.24	91406.7	516363899	8	
DNA-directed RNA polymerase subunit alpha	140.28	35032.5	516364804	14	
DNA-directed RNA polymerase subunit beta	150.28	133107.0	516364838	15	
DNA-directed RNA polymerase subunit beta	290.32	134729.4	516364837	29	
elongation factor P	280.27	76690.5	516364833	28	
elongation factor Ts	100.26	32339.5	516363753	10	
elongation factor Tu	198.27	43517.1	516364832	20	
histidyl-tRNA synthetase	30.21	48045.7	516365886	3	
arginyl-tRNA synthetase	40.24	62825.9	516366611	4	
asparaginyl-tRNA synthase	50.22	49068.6	516365513	5	
chromosomal replication initiation protein	40.23	51223.2	516366725	4	
alanyl-tRNA synthase	60.26	98169.4	516365872	6	
aspartyl/glutamyl-tRNA amidotransferase subunit A	20.19	52978.8	516364475	2	
aspartyl/glutamyl-tRNA amidotransferase subunit B	70.26	53068.1	516364474	7	
Amino acid metabolitic enzymes [Bacillus sp. ZYK]					
homoserine dehydrogenase	30.27	47109.1	516366270	3	
hydrogenase 2 large subunit	20.27	63660.9	516365215	2	
glucosamine--fructose-6-phosphate aminotransferase	20.18	66200.4	516364695	2	
glutamate dehydrogenase	60.25	46556.2	516365578	6	
glutamate-1-semialdehyde aminotransferase	30.25	46576.0	516365953	3	
glutamine amidotransferase	30.19	21520.8	516366737	3	
glutamine synthetase	70.28	50283.0	516363814	7	
glycine dehydrogenase subunit 2	30.21	54121.4	516365717	3	
cysteine desulfurase	78.36	44763.7	516366289	8	
cysteine synthase	130.25	32819.4	516364908	13	
delta-aminolevulinic acid dehydratase	18.25	36366.4	516365954	2	
dihydrolipoamide dehydrogenase	40.28	50901.3	516365674	4	
dihydroxynaphthoic acid synthetase	20.19	30009.1	516366166	2	
dipeptidase PepV	40.25	52350.1	648400774	4	
amidophosphoribosyltransferase	20.24	51419.0	516364489	2	
aminopeptidase T	30.24	45836.4	516363953	3	
aminotransferase	50.24	45871.0	516364305	5	
argininosuccinate lyase	18.23	51622.8	516366074	2	

argininosuccinate synthase	70.28	44472.6	516366075	7
aspartate aminotransferase	78.28	42976.0	516365514	8
aspartate ammonia-lyase	20.22	54022.0	516365979	2
aspartate-semialdehyde dehydrogenase	70.27	38208.5	516363778	7
alanine dehydrogenase	100.29	39733.7	516366072	10
branched-chain amino acid aminotransferase	70.25	39799.3	516365126	7
Others [Bacillus sp. ZYK]				
guanine permease	20.23	46267.9	516364969	2
heat shock protein 90	76.30	72495.1	516365355	8
heme peroxidase	98.26	28826.7	516366630	10
ethanolamine utilization protein EutJ	50.28	42289.8	516366677	5
Fe-S cluster assembly protein SufB	50.27	52561.4	516366287	5
flagellar biosynthesis protein FlhA	20.22	73971.9	516363738	2
flagellin	70.36	29454.4	516366410	7
cell division protein FtsH	60.28	72678.2	516364905	6
cell division protein FtsY	30.23	37015.1	516363664	3
cell division protein FtsZ	30.32	40686.8	516363606	3
chemotaxis protein CheA	150.28	75983.8	516363742	15
chemotaxis protein CheY	18.14	25731.3	516366430	2
chorismate synthase	30.23	42675.3	516365549	3
chromosome partitioning protein ParA	140.27	37832.6	516364794	14

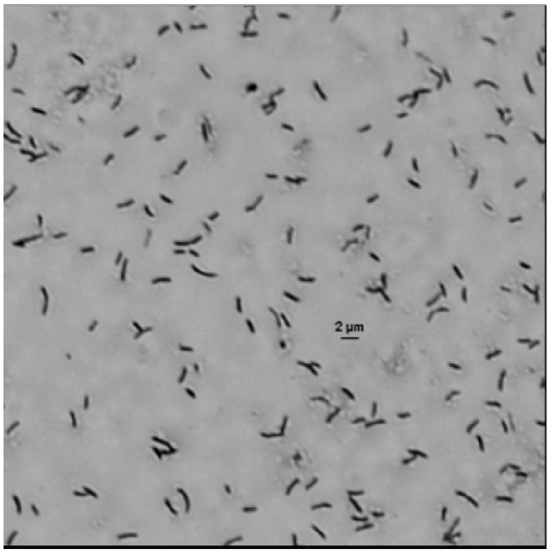

Figure S1. Phase-contrast light microscopy of strain ZYK^T. Scale bar corresponds to 2μm.

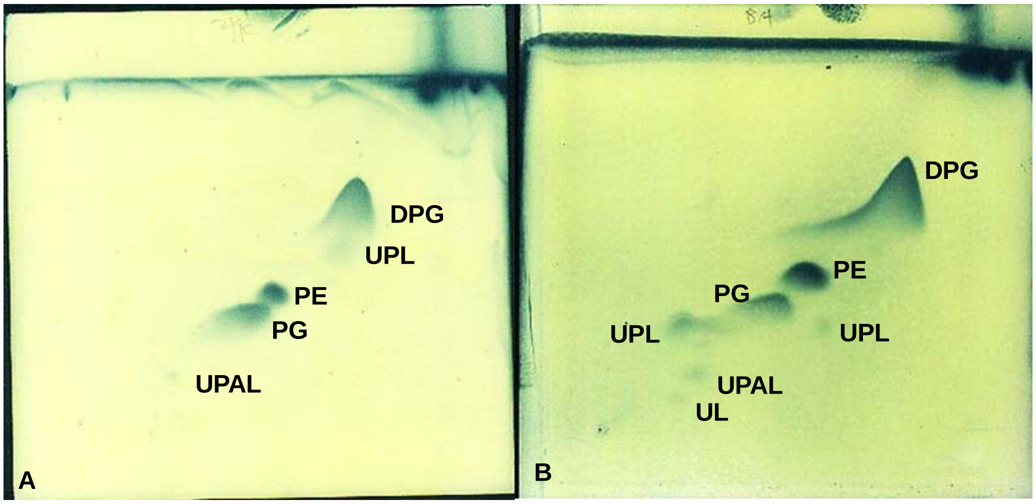

Figure S2. Thin layer chromatographic plates sprayed with molybdophosphoric acid reagents showing differential polar lipid compositions of strain ZYK^{T} (A) and *Bacillus azotoformans* ATCC 29788^{T} (B). DPG, diphosphatidylglycerol; PE, phosphatidylethanolamine; PG, phosphatidylglycerol; UPL, unknown phospholipid; UPAL, unidentified aminophospholipid; UL, unknown lipid.

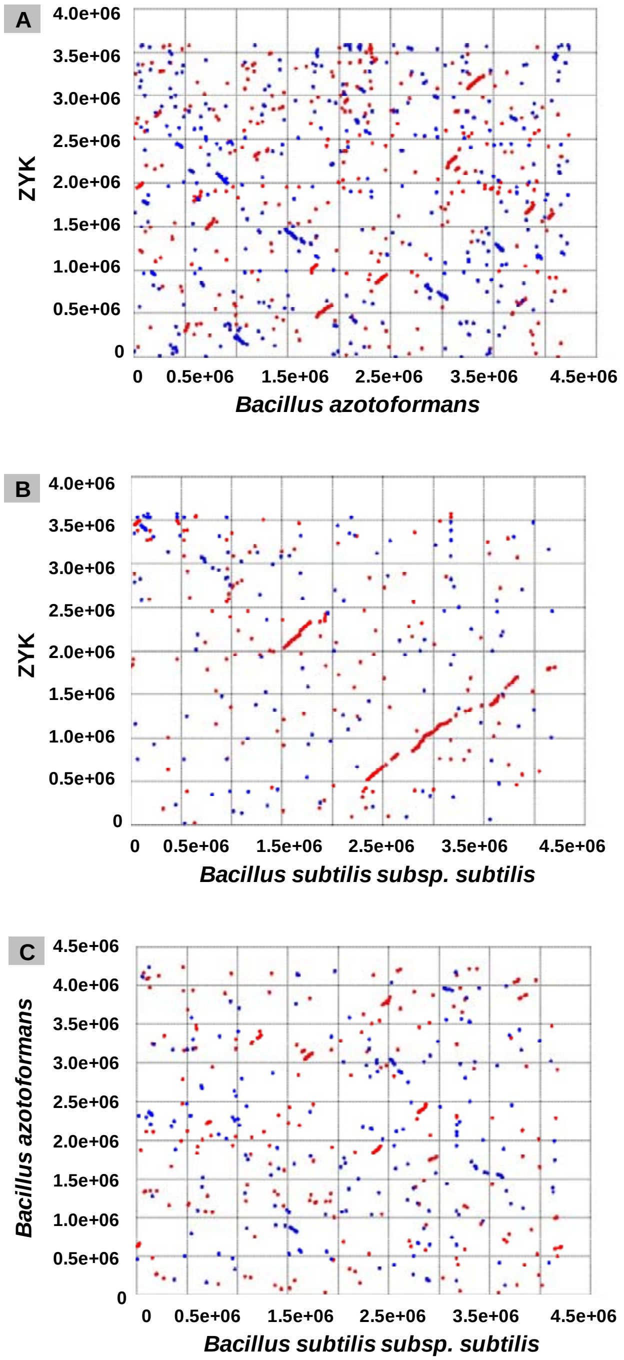

Figure S3. Dot plot representation of the pairwise alignments of the ZYK^T, *B. azotoformans* and *B. subtilis* subsp. *subtilis* genomes. A dot indicates a match (of at least six AA) between the two genome sequences being compared. The forward matches colored in red and reverse matches colored in blue.

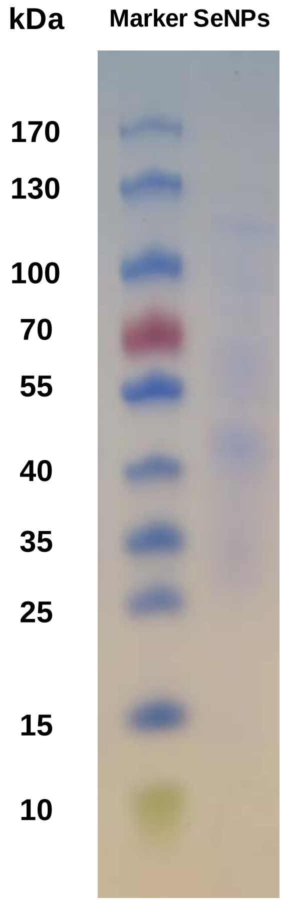

Figure S4. SDS-PAGE analysis of SeNPs high-affinity proteins.

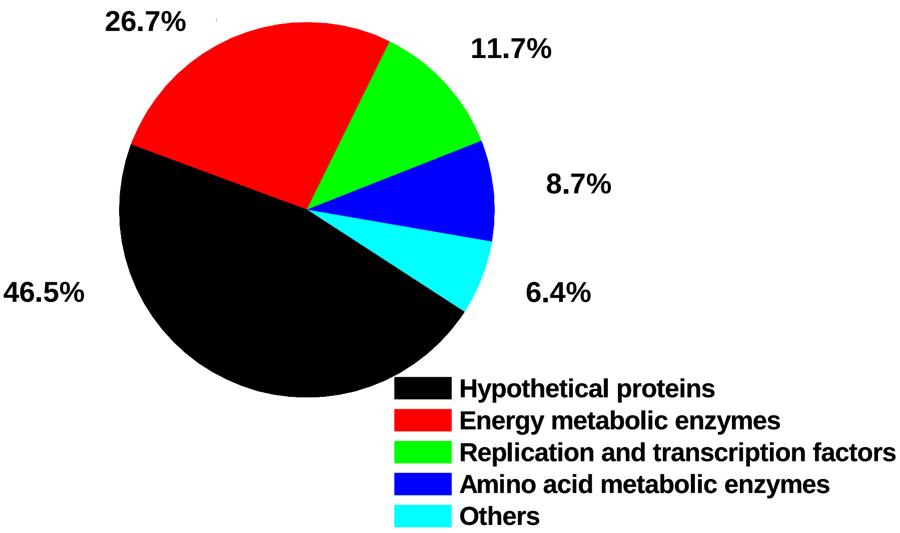

Figure S5. Relative proportions of SeNPs high-affinity proteins.
